# Cultural Adaptation and Validation of a Malay Version of the Craig Hospital Inventory of Environmental Factors (CHIEF) Among Older Adults in Malaysia

**DOI:** 10.7759/cureus.62999

**Published:** 2024-06-23

**Authors:** Nurul Syuhada Mohd Rosnu, Wan Syafira Ishak, Mohd Harimi Abd Rahman, Devinder Kaur Ajit Singh

**Affiliations:** 1 Centre for Healthy Ageing and Wellness (H-Care), Universiti Kebangsaan Malaysia, Kuala Lumpur, MYS; 2 Physiotherapy Program, Institut Jantung Negara College, Kuala Lumpur, MYS; 3 Optometry and Vision Sciences Programme, Centre for Rehabilitation and Special Needs, Universiti Kebangsaan Malaysia, Kuala Lumpur, MYS

**Keywords:** validity assessment, social participation, cultural adaptation, older adults, environmental barriers

## Abstract

Background/aim: The Craig Hospital Inventory of Environmental Factors (CHIEF) is a tool designed to assess and quantify the impact of environmental factors on an individual's functioning and social participation. In this study, we aim to culturally adapt the CHIEF from its original English version into the Malay language (M-CHIEF) and examine its validity and reliability among older adults in Malaysia.

Methods: The original CHIEF was cross-culturally adapted into the Malay language following the published guidelines on cross-cultural adaptation of health questionnaires. Its content and convergent validity were assessed using the content validity index and correlation with participants’ gait speed, respectively. The reliability of M-CHIEF was assessed for its internal consistency using Cronbach’s coefficient alpha and Cohen’s kappa and its test-retest reliability was assessed using intraclass correlation coefficients (ICCs).

Results: The M-CHIEF was rated with excellent content validity with a scale-level content validity index (S-CVI) of 0.86. Its internal consistency was demonstrated to be high with Cronbach’s alpha of 0.84. The test-retest reliability at a two-week interval showed a stable score of the M-CHIEF and its subscales with an ICC value of 0.89.

Conclusions: The M-CHIEF is deemed relevant for use among Malay speakers. It can function as an instrument to quantify the environmental barriers of an individual while considering broad environmental factors including policy, physical/ structural, work/school, attitude/support, and services/assistance.

## Introduction

The World Health Organization (WHO) has proposed the International Classification of Functioning, Disability and Health (ICF) to describe the dynamic interactions of environmental factors on a person’s functioning and participation in various life activities. Environmental factors, as defined by the ICF, include the physical, social, and attitudinal elements that surround a person and can either facilitate or hinder their ability to engage fully in daily life. For example, previous research has shown that older adults who maintain regular social interactions have been associated with better social and emotional well-being [1], cognitive function [2], and quality of life [3].

Following the impact of environmental factors on functioning and disability, in alignment with the WHO’s perspective, the Craig Hospital Inventory of Environmental Factors (CHIEF) was developed. This instrument was designed to enable the quantification of various environmental factors and delineate them into five distinct subscales, namely, policies, physical/structural, work/school, attitude/support, and services/assistance [4]. In contrast to other existing instruments that often focus narrowly on architectural and physical aspects of environmental factors [5,6], the CHIEF subscales provide a more comprehensive perspective, covering a broader spectrum of environmental factors.

The original CHIEF has excellent test-retest reliability with an intraclass correlation coefficient (ICC) value of 0.93 and high internal consistency (Cronbach's a = 0.93) [4]. CHIEF has been widely used and cross-culturally adapted to various languages for assessing environmental barriers in different populations, such as Brazil [7], Italy [8], Korea [9], and Iran [10]. The robust validity and reliability achieved in these studies affirm that the translated versions are suitable for use in languages other than the original. It serves as a valuable tool for researchers, clinicians, and policymakers to understand and address environmental factors affecting individuals with disabilities or health conditions.

To date, there is no published research that assesses the validity and reliability of the CHIEF questionnaire for the Malaysian population. Other than being recognized as an official language in Malaysia, the Malay language is the tenth most popular language in the world mainly used in Malaysia, Brunei, Indonesia, Singapore, and Thailand. Hence, it is crucial to have a validated and reliable translated questionnaire to be used in this population. Moreover, the adaptation of an instrument is required when the targeted population has a different sociocultural background [11]. Thus, the purpose of the present study was to translate and culturally adapt the CHIEF into the Malay language and to evaluate its validity reliability and test-retest reliability among community-dwelling older adults in Malaysia.

## Materials and methods

Demographic information, encompassing age, gender, and education level, was gathered from all participants. Subsequently, each participant was tasked with completing both the CHIEF questionnaire and a gait speed test.

The CHIEF is a questionnaire designed to assess and quantify environmental factors that may impact an individual's functioning. Developed by Whiteneck et al. in 2004, the CHIEF questionnaire contains 25 questions that are structured into five distinct subscales, namely, policies, physical/structural, work/school, attitude/support, and services/assistance. The policy subscale examines the influence of rules, regulations, and guidelines on an individual's daily life, whereas the physical/structural subscale focuses on the built environment, including architectural aspects and physical infrastructure, and how they may present barriers or facilitators. The work/school subscale is intended to assess the impact of the work or school environment on an individual's functioning, considering factors such as accessibility and support. The attitude/support subscale explores societal attitudes and the level of support individuals receive, emphasizing the psychosocial aspects of the environment. The final subscale, which is the services/assistance, aims to examine the availability and adequacy of services and assistance that individuals may require for their daily activities.

The mobility of the older adults was measured using the 10-meter walk test (10mW) test to measure gait speed. Older adults were required to walk over a 10-meter distance at their usual pace. Evaluating gait speed is appropriate for clinical settings due to its ability to be quick, reliable, cost-effective, practical, feasible, and helpful in predicting important health outcomes for older adults. This makes it a valuable tool for identifying older persons who may be at risk of these events and to validate the CHIEF questionnaire.

The gait speed was evaluated using the 10mW test where older adults were required to walk over a 10-meter distance at their usual pace. The timing was measured for the intermediate 6 meters to consider the time taken for acceleration and deceleration. The Asian Working Group of Sarcopenia (AWGS) cutoff value was used to categorize older adults’ walking speed. The 10mW test is a valid and reliable tool to assess gait speed among older adults and have an ICC value of 0.93 [12].

Procedures

This study's ethics were reviewed and approved by the research ethics committee of Universiti Kebangsaan Malaysia (approval no. UKM PPI/111/8/JEP-2021-742) and conforms to the Helsinki Declaration. The participants were provided written, informed consent before participating in this study.

Phase 1: Translation and Cultural Adaption of the CHIEF Questionnaire

Permission to translate the original M-CHIEF was first obtained from the original author through email. The original CHIEF questionnaire was translated into Malay language using the cross-cultural adaption guideline. This is to preserve the quality and meaning of the translated version of the original version. The adaption process has five stages: (1) forward translation, (2) synthesis of the translation, (3) back translation (4) expert committee, and (5) test of the pre-final version [13].

The forward translation process was conducted by the first author (NSMR) and an English teacher, both of whom are native Malay speakers. Subsequently, in a collaborative meeting, the translators and investigators involved in the present study engaged in discussions to resolve discrepancies and reached a consensus to produce a unified version in Malay. Following this, a linguist, whose primary language was English and whose second language was Malay, executed the back-translation, generating a new version in English. The original wording of the questionnaire and the contents were blinded by the linguist. The back-translated version was then compared with the original questionnaire, and any discrepancies were resolved through a collaboration between the translator and NSMR. Then, a panel comprising a physiotherapist (NSMR) and university professors (WSI, MHAR, and DKAS) who were fluent in both languages and familiar with the process of cultural adaptation conducted a thorough review and comparison of the original CHIEF version with the back-translation. This evaluation focused on aspects such as clarity in the social context of Malaysia and the preservation of the original meanings. In instances where inaccuracies or inconsistencies were identified during the panel review, they were discussed, and consensus was reached. Subsequently, the panel integrates the forward translation, backward translation, and the original version to construct the pre-final Malay version of the CHIEF questionnaire (M-CHIEF).

The pre-final M-CHIEF was then piloted to 20 older adults to analyze for internal consistency and item correlation value (ITC) and ensure clarity of the questions. Items that have ITC less than 0.2 were amended for better clarity. For example, the words “community” were changed to “residential area,” “terrain” to “road surface,” and “climate” to “weather.” This administration aimed to ensure the clarity of the questionnaire being used in Phase 2.

Phase 2: Evaluation of Psychometric Properties (Validity and Reliability)

The validity of the M-CHIEF was assessed by its content and construct validity. A total of six clinical physiotherapists and one occupational therapist from private and government settings with at least five years of working experience were recruited to participate in the content validity assessment. The experts were instructed to assess the relevancy and clarity of each item and subscale by assigning scores to the items and subscales. For relevancy, the scale ranges from 1 (not relevant to the domain) to 4 (highly relevant to the domain), whereas the scale for clarity ranges from 1 (not clear at all) to 4 (very clear). The content validity index (CVI) was calculated based on the recommended formula [14]. An instrument is considered to have a good validity if the CVI value is at least 0.83 [15]. The experts were allotted a duration of two weeks to complete the assessment. The items in the CHIEF questionnaire were revised based on the findings from the content validity assessment.

To recruit participants, flyers were distributed at selected primary healthcare services and several Klang Valley communities. Older adults aged 60 years and above, who live in Klang Valley, are able to ambulate with or without assistive devices, and are able to understand the Malay language were recruited for this study. Older adults with documented major psychiatric illnesses or mental disorders were excluded from the study. The period of recruitment of participants was from November 2021 to July 2022.

The participants completed a demographic questionnaire and the refined pre-final version of the CHIEF with a subsample of 30 older adults completed the M-CHIEF again two weeks later to assess the test-retest reliability. For construct validity, all participants underwent a gait speed test.

Data analysis

All analyses were carried out on IBM SPSS Statistics for Mac, version 26.0 (released 2019, IBM Corp., Armonk, NY). Demographic data were analyzed using central tendency (mean), dispersion (standard deviation) for age, and frequency distribution were used to characterize the sex and level of education.

To measure the CVI for the overall scale (S-CVI), the average CVI of the item (I-CVI) by all experts was calculated [14]. For construct validity, Spearman's correlation was used to correlate the M-CHIEF scores with the gait speed. Meanwhile, to measure the reliability of the M-CHIEF, Cronbach’s alpha was used to analyze the total score of the scale, while Cohen’s kappa coefficient for both frequency and magnitude scores are in the ordinal scale. The test-retest reliability of M-CHIEF was assessed with intraclass correlation (ICC).

## Results

Descriptive statistics

A total of 119 community-dwelling older adults who were predominantly female (66.4%) and had a mean (SD) age of 67.51 (5.54) years participated in this study. The descriptive characteristics of the participants (n = 119), along with those of the subgroup participating in the test-retest reliability (n = 30), are shown in Table 1.

**Table 1:** Descriptive characteristics of the total participants and the subgroup involved in the test-retest analysis of the M-CHIEF M-CHIEF: Malay language version of Craig Hospital Inventory of Environmental Factors, AWGS: Asian Working Group of Sarcopenia

Descriptive variables	Participants	
	Total sample N = 119	Test-retest reliability subgroup N = 30
Age, years, mean (SD)	67.51 (5.54)	65.37 (3.68)
Gender, n (%)		
Men	40 (33.6)	12 (32.4)
Women	79 (66.4)	18 (48.6)
Education level, n (%)		
Lower education	23 (19.3)	1 (2.7)
Higher education	96 (80.7)	29 (97.3)
CHIEF scores, mean (SD)		
Policy subscale	0.47 (0.88)	0.37 (0.61)
Physical/structural subscale	0.86 (1.12)	0.60 (0.82)
Work/school subscale	0.51 (0.80)	0.46 (0.71)
Attitudes/support subscale	0.29 (0.50)	0.42 (0.54)
Services/assistance subscale	0.47 (0.71)	0.33 (0.45)
Total	0.52 (0.58)	0.40 (0.46)
Gait AWGS, n (%)		
Usual gait speed	44 (37)	-
Slower gait speed	75 (63)	-

Convergent validity

The relationship between older adults’ gait speed and their CHIEF scores were examined for construct validity using Spearman’s correlation coefficients. As demonstrated in Table 2, the physical and structural subscale, services and assistance subscale, and total CHIEF score were significantly correlated with gait speed (0.16-0.19).

**Table 2:** Correlation between gait speed and the CHIEF subscales and total score CHIEF: Craig Hospital Inventory of Environmental Factors

Scale/Subscales	Gait
CHIEF	-0.181*
Policies	-0.085
Physical and structural	-0.189*
Work and school	0.315
Attitudes and support	-0.040
Services and assistance	-0.161*

Content validity

The overall content validity index of the instrument using a conservative approach (universal agreement approach) revealed that the translated CHIEF has excellent relevancy and clarity (S-CVI: 0.98). Out of 26 items (Q26 = magnitude of the barrier), all items scored the highest relevancy (I-CVI score: 1) except for items Q6, Q17, and Q26 with an I-CVI score of 0.86. For the clarity of the questions, four items (Q6, Q17, Q18, and Q26) had an I-CVI score of 0.86 while the other scored the highest clarity.

Reliability

Table 3 shows the internal reliability of the Malay (MY) version of CHIEF and the versions from Italy (ITLY), Sweden (SE), and the original CHIEF (USA). The overall score for M-CHIEF showed good internal consistency with Cronbach’s alpha of 0.84. The M-CHIEF subscales also showed good internal consistency with Cronbach’s alpha ranging from 0.84 to 0.94.

**Table 3:** Internal consistency of the Malay (MY) version, Italy (ITLY), Swedish (SE), and the original (USA). CHIEF: Craig Hospital Inventory of Environmental Factors

Scales and subscales	MY	ITLY	SE	USA (Original)
CHIEF	0.84	0.93	0.79	0.93
Policies	0.84	0.83	0.37	0.77
Physical and structural	0.86	0.74	0.56	0.77
Work and school	0.94	0.78	-	0.81
Attitudes and support	0.87	0.79	0.6	0.79
Services and assistance	0.91	0.76	0.48	0.76

Table 4 presents the test-retest stability analysis and reports the ICC and paired t-test for the M-CHIEF. The M-CHIEF scale and subscales each showed a value higher than 0.75, which indicates good test-retest reliability. According to the paired t-test, no significant difference between test and retest scores was examined, which indicates the absence of any systematic changes in the test-retest scores.

**Table 4:** Mean (SD) for the test and retest score and ICC for the CHIEF subscales, M-CHIEF-SF, and total score SD: standard deviation, ICC: intraclass correlation coefficient, CHIEF: Craig Hospital Inventory of Environmental Factors, M-CHIEF-SF: Malay version of Craig Hospital Inventory of Environmental Factors

Scale (#items)	Mean (SD)		p-value*	Test-retest reliability (ICC)	Confidence interval 95%	
	Test	Retest			Lower limit	Upper limit
Policies subscale	0.37 (0.61)	0.40 (0.72)	0.687	0.78	0.59	0.89
Policies businesses (23)	0.30(0.60)	0.30 (0.60)	1.000	0.81	0.64	0.91
Policies employment/education (24)	0.33 (0.84)	0.30 (0.65)	0.712	0.79	0.61	0.90
Services community (22)	0.50 (1.48)	0.57 (1.61)	0.573	0.92	0.83	0.96
Policies government (25)	0.43 (0.90)	0.43 (0.84)	0.476	0.63	0.35	0.80
Physical/structural subscale	0.60 (0.82)	0.56 (0.66)	0.305	0.83	0.68	0.92
Design home (2)	0.67 (1.61)	0.67 (1.61)	1.000	0.74	0.52	0.87
Surroundings (6)	0.57 (0.82)	0.33 (0.48)	0.070	0.47	0.15	0.70
Design community (4)	0.20 (0.41)	0.23 (0.43)	0.326	0.91	0.81	0.95
Design work/school (3)	0.67 (1.16)	0.67 (0.58)	1.000	0.50	-2.39	0.99
Natural environment (5)	0.83 (1.49)	0.80 (1.61)	0.787	0.91	0.82	0.96
Technology (11)	1.00 (2.07)	0.83 (1.74)	0.433	0.82	0.66	0.91
Work/school subscale	0.46 (0.71)	0.50 (0.76)	0.685	0.94	0.72	0.99
Support work/school (19)	0.40 (0.55)	0.40( 0.55)	1.000	1.00	-1.15	0.98
Attitude work/school (16)	0.25 (0.50)	0.50 (0.58)	0.391	0.57	0.48	0.97
Help work/school (13)	0.60 (0.80)	0.80 (0.84)	0.374	0.87	0.31	0.99
Attitudes/support subscale	0.42 (0.54)	0.49 (0.54)	0.252	0.83	0.68	0.92
Support community (20)	0.27 (0.52)	0.37 (0.62)	0.326	0.54	0.23	0.75
Attitudes community (17)	0.53 (0.90)	0.63 (0.77)	0.375	0.74	0.52	0.87
Support home (18)	0.33 (0.61)	0.37 (0.62)	0.712	0.68	0.43	0.84
Attitudes home (15)	0.77 (1.17)	0.77 (1.17)	1.000	0.83	0.67	0.91
Discrimination (21)	0.20 (0.48)	0.30 (0.60)	0.264	0.61	0.33	0.79
Services/assistance subscale	0.33 (0.45)	0.36 (0.45)	0.396	0.92	0.84	0.96
Transportation (1)	0.07 (0.25)	0.07 (0.25)	1.000	1.00	-	-
Medical care (9)	0.53 (1.07)	0.50 (0.86)	0.801	0.73	0.51	0.86
Help home (12)	0.33 (0.48)	0.53 (0.86)	0.083	0.60	0.32	0.79
Education/training (8)	0.13 (0.35)	0.10 (0.31)	0.573	0.53	0.21	0.74
Help community (14)	0.37 (0.49)	0.47 (0.57)	0.184	0.71	0.48	0.85
Information (7)	0.63 (0.93)	0.57 (0.86)	0.423	0.88	0.76	0.94
Personal equipment (10)	0.23 (0.77)	0.27 (0.58)	0.745	0.68	0.42	0.83
CHIEF total	0.40 (0.46)	0.44 (0.48)	0.744	0.89	0.78	0.94

Table 5 shows the classification of the Kappa coefficients for each question of the CHIEF. Each question in the M-CHIEF was reproducible and ranged from “fair” to “excellent."

**Table 5:** Classification of the Kappa coefficients obtained for each question of the M-CHIEF (N = 30) in the test-retest reliability assessment for frequency and magnitude score M-CHIEF: Malay version of the Craig Hospital Inventory of Environmental Factors

Reference criteria for Kappa coefficient	Question frequency score	Question magnitude score
>0.80 (excellent)	Q1, Q4, Q17, Q23	Q1, Q4, Q15
0.60–0.80 (good)	Q3, Q5, Q6, Q7, Q10, Q12, Q13, Q14, Q15, Q16, Q18, Q19, Q20, Q22, Q24,	Q3, Q5, Q6, Q7, Q10, Q11, Q12, Q13, Q14, Q16, Q17, Q22, Q23, Q24
0.40–0.60 (fair)	Q2, Q8, Q9, Q11, Q21, Q25	Q2, Q8, Q9, Q18, Q19, Q20, Q21, Q25
<0.40 (poor)		

## Discussion

This study aimed to adapt and validate the CHIEF questionnaire for use in a population that has a different culture and language from the original population where the CHIEF was developed. The translation and back-translation processes in this study resulted in the development of M-CHIEF, which may be used among Malaysians. The translators involved were highly competent in both English and Malay languages and well-versed with the targeted population culture. This is crucial to ensure the appropriateness of the translation outcome. Following the translation and adaptation process, the M-CHIEF was administered to older adults without serious or disabling pathologies.

Clinical physiotherapists and clinical occupational therapists were selected to validate the contents of the M-CHIEF. This is crucial to ensure the M-CHIEF is relevant and can be accepted in clinical settings, apart from research settings. The overall M-CHIEF revealed excellent relevancy and clarity (S-CVI: 0.98). Most of the items scored the highest relevancy and clarity except for Q6, Q17, and Q18 and the magnitude of the barrier. For items Q6, Q17, and Q18, experts suggest further elaboration or example is needed to improve the item clarity. For instance, in item Q6, the type of information was deemed needed to be clarified for better clarity. However, in our final M-CHIEF, no further elaboration or example was added. To improve clarity and eliminate any difficulties, the researcher or administrator may assist in giving verbal examples to the respondents. This is also shared by Furtado et al. (2014), where some items require verbal examples to facilitate respondents’ understanding.

The M-CHIEF was also analyzed for convergent validity with gait speed that measures how the gait speed correlates with the M-CHIEF. Our study findings indicate that the total CHIEF score, physical and structural subscale, services, and assistance subscale were significantly correlated with gait speed (0.16-0.19). This is expected as the correlation between environment and gait speed among older adults has been demonstrated in the existing literature. In the Malaysian context, built environment characteristics play a significant external factor in the maintenance of older adults’ functional mobility, such as gait speed [16]. The CHIEF has also been correlated with activities of daily living (ADL) among Korean community-dwelling older adults, which indicates that older adults with lower ADL reported higher environmental barriers [17].

The M-CHIEF reliability was analyzed using Cronbach’s alpha, Cohen’s kappa, and test-retest reliability. The result of this study shows that the overall M-CHIEF and all subscales have a good internal consistency with Cronbach’s alpha >0.70. Cohen’s kappa was used to analyze the internal consistency of the frequency and magnitude of each question. For the test-retest reliability, the overall M-CHIEF and each subscale showed excellent stability with an ICC value >0.70. The overall M-CHIEF score and each subscale retains excellent stability after repeated administration to the same participants, at two weeks' interval time. This excellent reliability of the M-CHIEF is in line with the original version and other versions [7,10,18]. With an ICC value of 0.78, the policy subscale of the M-CHIEF appears as the least stable among all the subscales. This is consistent with the Italian version of the CHIEF scale [8].

This study has its limitations. It was done among specific age groups without serious or disabling pathologies. For future studies, we suggest that the M-CHIEF should be tested for validity and reliability with other environment-specific instruments to prove the strength of the construct validity of the M-CHIEF. In addition, future studies may also cross-validate the M-CHIEF with other age groups and among people with disabilities or specific needs, such as wheelchair users.

## Conclusions

We examined the validity and utility of the CHIEF among Malay-speaking older adults in Malaysia. This study is the first to examine and analyze the Malay version of the CHIEF’s psychometric properties. We believe that the M-CHIEF may provide a wide understanding of the influence of environmental barriers on individuals' function and social participation among Malay speakers. Although the M-CHIEF was validated among specific age groups, we believe that the M-CHIEF could be used by the general population. There are limited existing environmental barrier measurements available. The five subscales of the M-CHIEF potentially have clinical and political important implications. Having a validated instrument that covers various aspects of the environmental barriers in Malaysia's social and cultural context may facilitate rehabilitation clinicians and policy makers to identify and elevate the barriers.
